# p53 and ovarian carcinoma survival: an Ovarian Tumor Tissue Analysis consortium study

**DOI:** 10.1002/cjp2.311

**Published:** 2023-03-22

**Authors:** Martin Köbel, Eun‐Young Kang, Ashley Weir, Peter F Rambau, Cheng‐Han Lee, Gregg S Nelson, Prafull Ghatage, Nicola S Meagher, Marjorie J Riggan, Jennifer Alsop, Michael S Anglesio, Matthias W Beckmann, Christiani Bisinotto, Michelle Boisen, Jessica Boros, Alison H Brand, Angela Brooks‐Wilson, Michael E Carney, Penny Coulson, Madeleine Courtney‐Brooks, Kara L Cushing‐Haugen, Cezary Cybulski, Suha Deen, Mona A El‐Bahrawy, Esther Elishaev, Ramona Erber, Sian Fereday, AOCS Group, Anna Fischer, Simon A Gayther, Arantzazu Barquin‐Garcia, Aleksandra Gentry‐Maharaj, C Blake Gilks, Helena Gronwald, Marcel Grube, Paul R Harnett, Holly R Harris, Andreas D Hartkopf, Arndt Hartmann, Alexander Hein, Joy Hendley, Brenda Y Hernandez, Yajue Huang, Anna Jakubowska, Mercedes Jimenez‐Linan, Michael E Jones, Catherine J Kennedy, Tomasz Kluz, Jennifer M Koziak, Jaime Lesnock, Jenny Lester, Jan Lubiński, Teri A Longacre, Maria Lycke, Constantina Mateoiu, Bryan M McCauley, Valerie McGuire, Britta Ney, Alexander Olawaiye, Sandra Orsulic, Ana Osorio, Luis Paz‐Ares, Teresa Ramón y Cajal, Joseph H Rothstein, Matthias Ruebner, Minouk J Schoemaker, Mitul Shah, Raghwa Sharma, Mark E Sherman, Yurii B Shvetsov, Naveena Singh, Helen Steed, Sarah J Storr, Aline Talhouk, Nadia Traficante, Chen Wang, Alice S Whittemore, Martin Widschwendter, Lynne R Wilkens, Stacey J Winham, Javier Benitez, Andrew Berchuck, David D Bowtell, Francisco J Candido dos Reis, Ian Campbell, Linda S Cook, Anna DeFazio, Jennifer A Doherty, Peter A Fasching, Renée T Fortner, María J García, Marc T Goodman, Ellen L Goode, Jacek Gronwald, David G Huntsman, Beth Y Karlan, Linda E Kelemen, Stefan Kommoss, Nhu D Le, Stewart G Martin, Usha Menon, Francesmary Modugno, Paul DP Pharoah, Joellen M Schildkraut, Weiva Sieh, Annette Staebler, Karin Sundfeldt, Anthony J Swerdlow, Susan J Ramus, James D Brenton

**Affiliations:** ^1^ Department of Pathology and Laboratory Medicine University of Calgary, Foothills Medical Center Calgary AB Canada; ^2^ School of Clinical Medicine UNSW Medicine and Health, University of NSW Sydney Sydney New South Wales Australia; ^3^ Adult Cancer Program, Lowy Cancer Research Centre University of NSW Sydney Sydney New South Wales Australia; ^4^ The Walter and Eliza Hall Institute of Medical Research Parkville Victoria Australia; ^5^ Pathology Department Catholic University of Health and Allied Sciences‐Bugando Mwanza Tanzania; ^6^ Department of Pathology and Laboratory Medicine University of Alberta Edmonton AB Canada; ^7^ Department of Oncology, Division of Gynecologic Oncology, Cumming School of Medicine University of Calgary Calgary AB Canada; ^8^ The Daffodil Centre The University of Sydney, a Joint Venture with Cancer Council NSW Sydney New South Wales Australia; ^9^ Department of Obstetrics and Gynecology, Division of Gynecologic Oncology Duke University Medical Center Durham NC USA; ^10^ Centre for Cancer Genetic Epidemiology, Department of Oncology University of Cambridge Cambridge UK; ^11^ Department of Obstetrics and Gynecology University of British Columbia Vancouver BC Canada; ^12^ British Columbia's Gynecological Cancer Research Team (OVCARE) University of British Columbia, BC Cancer, and Vancouver General Hospital Vancouver BC Canada; ^13^ Department of Gynecology and Obstetrics, Comprehensive Cancer Center Erlangen‐EMN Friedrich‐Alexander University Erlangen‐Nuremberg, University Hospital Erlangen Erlangen Germany; ^14^ Department of Gynecology and Obstetrics, Ribeirão Preto Medical School University of São Paulo Ribeirão Preto Brazil; ^15^ Division of Gynecologic Oncology, Department of Obstetrics, Gynecology and Reproductive Sciences University of Pittsburgh School of Medicine Pittsburgh PA USA; ^16^ Centre for Cancer Research The Westmead Institute for Medical Research, University of Sydney Sydney New South Wales Australia; ^17^ Department of Gynaecological Oncology Westmead Hospital Sydney New South Wales Australia; ^18^ Discipline of Obstetrics and Gynaecology The University of Sydney Sydney New South Wales Australia; ^19^ Canada's Michael Smith Genome Sciences Centre, BC Cancer Vancouver BC Canada; ^20^ Department of Obstetrics and Gynecology, John A. Burns School of Medicine University of Hawaii Honolulu HI USA; ^21^ Division of Genetics and Epidemiology The Institute of Cancer Research London UK; ^22^ Program in Epidemiology, Division of Public Health Sciences, Fred Hutchinson Cancer Research Center Seattle WA USA; ^23^ Department of Genetics and Pathology, International Hereditary Cancer Center Pomeranian Medical University Szczecin Poland; ^24^ Department of Histopathology Nottingham University Hospitals NHS Trust, Queen's Medical Centre Nottingham UK; ^25^ Department of Metabolism, Digestion and Reproduction Imperial College London, Hammersmith Hospital London UK; ^26^ Department of Pathology University of Pittsburgh School of Medicine Pittsburgh PA USA; ^27^ Institute of Pathology, Comprehensive Cancer Center Erlangen‐EMN, Friedrich‐Alexander University Erlangen‐Nuremberg, University Hospital Erlangen Erlangen Germany; ^28^ Peter MacCallum Cancer Centre Melbourne Victoria Australia; ^29^ Sir Peter MacCallum Department of Oncology The University of Melbourne Parkville Victoria Australia; ^30^ QIMR Berghofer Medical Research Institute Brisbane Queensland Australia; ^31^ Institute of Pathology and Neuropathology, Tuebingen University Hospital Tuebingen Germany; ^32^ Center for Bioinformatics and Functional Genomics and the Cedars Sinai Genomics Core, Cedars‐Sinai Medical Center Los Angeles CA USA; ^33^ HM Sanchinarro Centro Integral Oncológico Clara Campal University Hospital Madrid Spain; ^34^ MRC Clinical Trials Unit Institute of Clinical Trials & Methodology, University College London London UK; ^35^ Department of Pathology and Laboratory Medicine University of British Columbia Vancouver BC Canada; ^36^ Department of Propaedeutics, Physical Diagnostics and Dental Physiotherapy Pomeranian Medical University Szczecin Poland; ^37^ Department of Women's Health Tuebingen University Hospital Tuebingen Germany; ^38^ Crown Princess Mary Cancer Centre Westmead Hospital Sydney New South Wales Australia; ^39^ Department of Epidemiology University of Washington Seattle WA USA; ^40^ Department of Gynecology and Obstetrics University Hospital of Ulm Ulm Germany; ^41^ Cancer Epidemiology Program University of Hawaii Cancer Center Honolulu HI USA; ^42^ Department of Laboratory Medicine and Pathology, Mayo Clinic Rochester MN USA; ^43^ Independent Laboratory of Molecular Biology and Genetic Diagnostics Pomeranian Medical University Szczecin Poland; ^44^ Department of Histopathology Addenbrooke's Hospital Cambridge UK; ^45^ Department of Gynecology and Obstetrics Institute of Medical Sciences, Medical College of Rzeszow University Rzeszów Poland; ^46^ Alberta Health Services‐Cancer Care Calgary AB Canada; ^47^ David Geffen School of Medicine, Department of Obstetrics and Gynecology University of California at Los Angeles Los Angeles CA USA; ^48^ Department of Pathology Stanford University School of Medicine Stanford CA USA; ^49^ Department of Obstetrics and Gynecology Institute of Clinical Science, Sahlgrenska University Hospital, University of Gothenburg Gothenburg Sweden; ^50^ Department of Pathology University of Gothenburg Gothenburg Sweden; ^51^ Department of Quantitative Health Sciences, Division of Epidemiology, Mayo Clinic Rochester MN USA; ^52^ Department of Epidemiology and Population Health Stanford University School of Medicine Stanford CA USA; ^53^ Genetics Service, Fundación Jiménez Díaz Madrid Spain; ^54^ Centre for Biomedical Network Research on Rare Diseases (CIBERER) Instituto de Salud Carlos III Madrid Spain; ^55^ H12O‐CNIO Lung Cancer Clinical Research Unit, Spanish National Cancer Research Centre (CNIO) Madrid Spain; ^56^ Oncology Department Hospital Universitario 12 de Octubre Madrid Spain; ^57^ Medical Oncology Service Hospital Sant Pau Barcelona Spain; ^58^ Department of Genetics and Genomic Sciences Icahn School of Medicine at Mount Sinai New York NY USA; ^59^ Department of Population Health Science and Policy Icahn School of Medicine at Mount Sinai New York NY USA; ^60^ Tissue Pathology and Diagnostic Oncology Westmead Hospital Sydney New South Wales Australia; ^61^ Department of Health Sciences Research, Mayo Clinic Jacksonville FL USA; ^62^ Division of Gynecologic Oncology, Department of Obstetrics and Gynecology University of Alberta Edmonton AB Canada; ^63^ Section of Gynecologic Oncology Surgery, North Zone, Alberta Health Services Edmonton AB Canada; ^64^ Nottingham Breast Cancer Research Centre Biodiscovery Institute, University of Nottingham Nottingham UK; ^65^ Department of Quantitative Health Sciences, Division of Computational Biology, Mayo Clinic Rochester MN USA; ^66^ Department of Biomedical Data Science Stanford University School of Medicine Stanford CA USA; ^67^ EUTOPS Institute, University of Innsbruck Innsbruck Austria; ^68^ Human Genetics Group, Spanish National Cancer Research Centre (CNIO) Madrid Spain; ^69^ Epidemiology, School of Public Health University of Colorado Aurora CO USA; ^70^ Community Health Sciences, University of Calgary Calgary AB Canada; ^71^ Huntsman Cancer Institute, Department of Population Health Sciences University of Utah Salt Lake City UT USA; ^72^ Division of Cancer Epidemiology, German Cancer Research Center (DKFZ) Heidelberg Germany; ^73^ Department of Research, Cancer Registry of Norway Oslo Norway; ^74^ Computational Oncology Group, Structural Biology Programme, Spanish National Cancer Research Centre (CNIO) Madrid Spain; ^75^ Cancer Prevention and Control Program, Cedars‐Sinai Cancer, Cedars‐Sinai Medical Center Los Angeles CA USA; ^76^ Department of Molecular Oncology, BC Cancer Research Centre Vancouver BC Canada; ^77^ Division of Acute Disease Epidemiology, South Carolina Department of Health & Environmental Control Columbia SC USA; ^78^ Cancer Control Research, BC Cancer Agency Vancouver BC Canada; ^79^ Department of Epidemiology University of Pittsburgh School of Public Health Pittsburgh PA USA; ^80^ Women's Cancer Research Center Magee‐Womens Research Institute and Hillman Cancer Center Pittsburgh PA USA; ^81^ Department of Computational Biomedicine, Cedars‐Sinai Medical Center West Hollywood CA USA; ^82^ Centre for Cancer Genetic Epidemiology, Department of Public Health and Primary Care University of Cambridge Cambridge UK; ^83^ Department of Epidemiology, Rollins School of Public Health Emory University Atlanta GA USA; ^84^ Department of Obstetrics and Gynecology, Institute of Clinical Science Sahlgrenska Center for Cancer Research, University of Gothenburg Gothenburg Sweden; ^85^ Division of Breast Cancer Research The Institute of Cancer Research London UK; ^86^ Cancer Research UK Cambridge Institute, University of Cambridge Cambridge UK

**Keywords:** ovarian cancer, high‐grade serous carcinoma, endometrioid, clear cell, *TP53*, p53, prognosis

## Abstract

Our objective was to test whether p53 expression status is associated with survival for women diagnosed with the most common ovarian carcinoma histotypes (high‐grade serous carcinoma [HGSC], endometrioid carcinoma [EC], and clear cell carcinoma [CCC]) using a large multi‐institutional cohort from the Ovarian Tumor Tissue Analysis (OTTA) consortium. p53 expression was assessed on 6,678 cases represented on tissue microarrays from 25 participating OTTA study sites using a previously validated immunohistochemical (IHC) assay as a surrogate for the presence and functional effect of *TP53* mutations. Three abnormal expression patterns (overexpression, complete absence, and cytoplasmic) and the normal (wild type) pattern were recorded. Survival analyses were performed by histotype. The frequency of abnormal p53 expression was 93.4% (4,630/4,957) in HGSC compared to 11.9% (116/973) in EC and 11.5% (86/748) in CCC. In HGSC, there were no differences in overall survival across the abnormal p53 expression patterns. However, in EC and CCC, abnormal p53 expression was associated with an increased risk of death for women diagnosed with EC in multivariate analysis compared to normal p53 as the reference (hazard ratio [HR] = 2.18, 95% confidence interval [CI] 1.36–3.47, *p* = 0.0011) and with CCC (HR = 1.57, 95% CI 1.11–2.22, *p* = 0.012). Abnormal p53 was also associated with shorter overall survival in The International Federation of Gynecology and Obstetrics stage I/II EC and CCC. Our study provides further evidence that functional groups of *TP53* mutations assessed by abnormal surrogate p53 IHC patterns are not associated with survival in HGSC. In contrast, we validate that abnormal p53 IHC is a strong independent prognostic marker for EC and demonstrate for the first time an independent prognostic association of abnormal p53 IHC with overall survival in patients with CCC.

## Introduction

*TP53* is universally mutated in tubo‐ovarian high‐grade serous carcinomas (HGSCs), and a lack of *TP53* mutation is essentially inconsistent with a diagnosis of HGSC [1, 2]. *TP53* mutations are broadly classified into gain‐of‐function (GOF) mutations (mutation type: nonsynonymous/missense, which are further subclassified into contact and conformational) versus loss‐of‐function (LOF) mutations (mutation type: frameshift [insertion/deletions], stop‐gain, and splicing). We have recently optimised p53 immunohistochemistry (IHC) to serve as an accurate surrogate for the *TP53* mutation status with 96% sensitivity and perfect specificity [3]. The high, but not perfect, sensitivity was influenced by non‐functional p53 proteins caused by late truncating *TP53* mutations that showed normal wild type pattern by IHC [3]. The abnormal patterns of p53 IHC also showed good agreement with the function of the mutation: overexpression (OE) for nonsynonymous GOF, and complete absence (CA) for LOF, the latter with some explainable exceptions [3]. The uncommon abnormal cytoplasmic (CY) pattern is associated with *TP53* mutations in the nuclear localisation domain, adjacent to the tetramerisation domain or nuclear exclusion sequence [3, 4].

The prognostic associations of specific mutation types in HGSC have been controversial. Using p53 IHC, we previously reported that the LOF surrogate CA was associated with shorter survival in a combined cohort of 502 HGSC [5]. However, using *TP53* mutation data from The Cancer Genome Atlas (TCGA) project, Kang *et al* showed no difference between GOF and other *TP53* mutations with respect to overall survival [6]. Using a different classification in the TCGA data set, Brachova *et al* reported a higher risk of recurrence for GOF mutations [7]. More recently, Mandilaras *et al* showed no difference in overall survival using six different classification schemas for *TP53* mutations; however, one cluster enriched in GOF mutations was associated with a worse prognosis [8]. Tuna *et al* reported that three hot‐spot GOF mutations (at G266, Y163, R282) were associated with shorter overall survival, but they did not find differences for different types of *TP53* mutations [9].

In contrast to HGSC, only subsets of endometrioid carcinoma (EC) and clear cell carcinoma (CCC) harbour *TP53* mutations [5]. Like its endometrial counterpart, in ovarian EC, abnormal p53 expression by IHC defines a prognostically adverse molecular subtype according to three studies totalling 749 cases [10, 11, 12]. However, the prognostic value in the low‐stage setting (The International Federation of Gynecology and Obstetrics [FIGO] stage I/II) has only been addressed in one study of 274 cases [11]. In CCC, two studies with 90 and 115 cases reported associations of abnormal p53 status assessed by IHC with shorter survival in univariate but not multivariate analysis [13, 14]. Larger genomic studies of 271 and 421 CCC showed associations of *TP53* mutations with gene expression and methylation clusters that tend to show adverse clinical features but did not report an independent prognostic value for *TP53* alone [15, 16]. Abnormal p53 function in endometrial carcinoma is associated with chromosomal instability (CIN) and adverse outcome and is now a strong indication for more aggressive management including adjuvant chemotherapy [17]. Accurate assessment of the impacts of abnormal p53 staining on outcome may be clinically important in low‐stage EC and CCC. The aims of the current study were to (1) clarify whether types of *TP53* mutations inferred by three abnormal p53 IHC patterns are associated with prognosis in HGSC; (2) validate whether abnormal p53 IHC predicts higher risk of death in EC, particularly for FIGO stage I and II disease; and (3) elucidate the association of p53 status with overall survival in CCC using a large cohort from the Ovarian Tumor Tissue Analysis (OTTA) consortium [18, 19, 20].

## Methods

### Study cohort

Twenty‐five studies from the OTTA consortium contributed to this study. Each participating study received local ethics board approval (supplementary material, Table S1). Cases included in this study were recruited before the widespread use of diagnostic IHC (median year of diagnosis 2004, 25–75% quartiles 2000–2008, range 1978–2016). The 4‐μm‐thick sections of previously constructed tissue microarrays with each case represented by 1–3 cores were shipped to a central immunohistochemical laboratory at the University of Calgary, Alberta, Canada. Clinical covariates and follow‐up time and status were centrally collected at the University of New South Wales, Sydney, Australia. Histotype was assessed by various tiers of pathology review based on the 2014 World Health Organization (WHO) classification [21] (supplementary material, Table S1). Since the histotype review was heterogeneous, WT1 alone or in combination with p53 IHC was further used to identify potentially misclassified cases. Based on previous data that HGSC typically shows a combination of WT1 expression and abnormal p53 versus <1% of CCC and <2% of EC [22], we reassigned 23 WT1+/p53‐abnormal EC to HGSC, excluded 30 WT1+ CCC as they likely represent HGSC or low‐grade serous carcinoma, and excluded 121 WT1−/p53‐normal HGSC. Previously generated data and categorisation, where applicable, were used for CD8+ tumour infiltrating lymphocytes (TILs), CDKN2A, and *TP53* mRNA expression [19, 20, 23].

### Immunohistochemistry

Two previously validated p53 IHC assays were used [3, 24]. The first assay (pre‐treatment with BOND Epitope Retrieval Solution 2, Leica Biosystems, Wetzlar, Germany; p53 antibody clone DO‐7, dilution 1:2,500, Dako Omnis, Agilent, Santa Clara, CA, USA) was used to stain 55.4% of cases and 44.6% of cases were stained with a second assay (pre‐treatment for 30 min using heat‐induced antigen retrieval with Tris‐EDTA buffer, pH = 9.0; ready‐to‐use antibody DO‐7, Dako Omnis) due to a change in the automated IHC platform during the study period. The interpretation criteria for the three abnormal patterns were defined as follows: OE as strong nuclear staining of similar intensity in usually all but at least 80% of tumour cell nuclei; CA as complete absence of nuclear staining with retained staining in normal fibroblasts or lymphocytes serving as internal controls; and CY as unequivocal cytoplasmic staining of at least of moderate intensity and absent or variate nuclear staining [25]. In contrast, the normal (wild type) pattern was characterised by staining of variate intensity in tumour nuclei. After assessing the interobserver agreement with a kappa coefficient of 0.912 on a test set of *n* = 92 cases, one observer (MK) scored approximately 75% and a second observer (PR) scored 25% of the cases.

### Statistical analyses

The distributions of categorical or continuous variables were compared across histotypes using chi‐square or ANOVA tests, respectively. Time from diagnosis to death, due to any cause, was the primary endpoint. Left truncation was applied to mitigate potential survival bias introduced by the time between diagnosis and study enrolment and right censoring at 10 years from diagnosis was used to account for potential non‐cancer‐related deaths. Univariate Kaplan‐Meier survival analyses alongside log‐rank testing were used to assess overall survival. Cox proportional hazards regression models were applied to estimate hazard ratios (HRs) with 95% confidence intervals (CIs). Univariate Cox regression models evaluated associations with overall survival for covariates, including age (continuous), FIGO stage (III/IV versus reference I/II), the completeness of surgical cytoreduction (residual disease present versu*s* absent/unknown), p53 status (normal versus abnormal) and, where applicable, grade (only appropriate for EC: 3 versus reference 1/2). Multivariate Cox regression models were adjusted for age (continuous), FIGO stage (III/IV versus reference I/II), grade (only appropriate for EC: 3 versus reference 1/2), the completeness of surgical cytoreduction (residual disease present versus absent/unknown), and stratified by the OTTA study contributing the sample to account for differing baseline hazards. Statistical analyses were carried out using SAS JMPv16.2.0 or RStudio v1.1.463. Statistical significance was defined by *p* < 0.05.

## Results

### Baseline clinicopathological features of study participants

The analysis included 6,678 patients with one of the three most common ovarian carcinoma histotypes (HGSC, EC, and CCC). By histotype, the 4,957 patients with HGSC were significantly older (mean age 60.2 years) compared to the 973 patients with EC (54.4 years) and 748 patients with CCC (55.3 years; *p* < 0.001; Table 1). Expected differences were observed for stage, residual disease, and 5‐year survival rate (5‐YSR) across histotypes. For example, 80.9% of HGSC were diagnosed at high stage (III or IV) versus only 14.1% of EC. The 5‐YSR was highest for patients diagnosed with EC (84.4%), intermediate for patients diagnosed with CCC (66.2%), and lowest for patients with HGSC (41.7%) (*p* < 0.001; Table 1).

**Table 1:** Clinicopathological characteristics and p53 expression patterns across histotypes

		HGSC	%	EC	%	CCC	%	Total	%	*P* value
*n*	Total	4,957		973		748		6,678		
Age	Mean	60.2		54.4		55.3		58.8		<0.001
	Min–Max	22–93		21–91		27–84		21–93		
FIGO stage	I/II	894	19.1	771	85.9	550	76.6	2,215	35.2	<0.001
	III/IV	3,775	80.9	127	14.1	168	23.4	4,070	64.8	
	Unknown	288		75		30		393		
Residual disease	Absent	893	37.8	422	89.0	324	79.0	1,639	50.4	<0.001
	Present	1,472	62.2	52	11.0	86	21.0	1,610	49.6	
	Unknown	2,592		499		338		3,429		
5‐YSR	%, SE	41.7, 0.8		84.4, 1.2		66.2, 1.8		50.8, 0.6		<0.001
	Unknown	200		41		23		264		
p53	Normal	327	6.6	857	88.1	662	88.5	1,846	27.6	<0.001
	Abnormal	4,630	93.4	116	11.9	86	11.5	4,832	72.4	
	Abnormal OE	3,221	69.6	85	73.3	68	79.1	3,374	69.8	0.23
	Abnormal CA	1,176	25.4	27	23.3	17	19.8	1,220	25.2	
	Abnormal CY	233	5.0	4	3.4	1	1.2	238	4.9	

*P* values calculated excluding cases with ‘unknown’ information.

### Prevalence of abnormal p53 IHC

**Figure 1:**
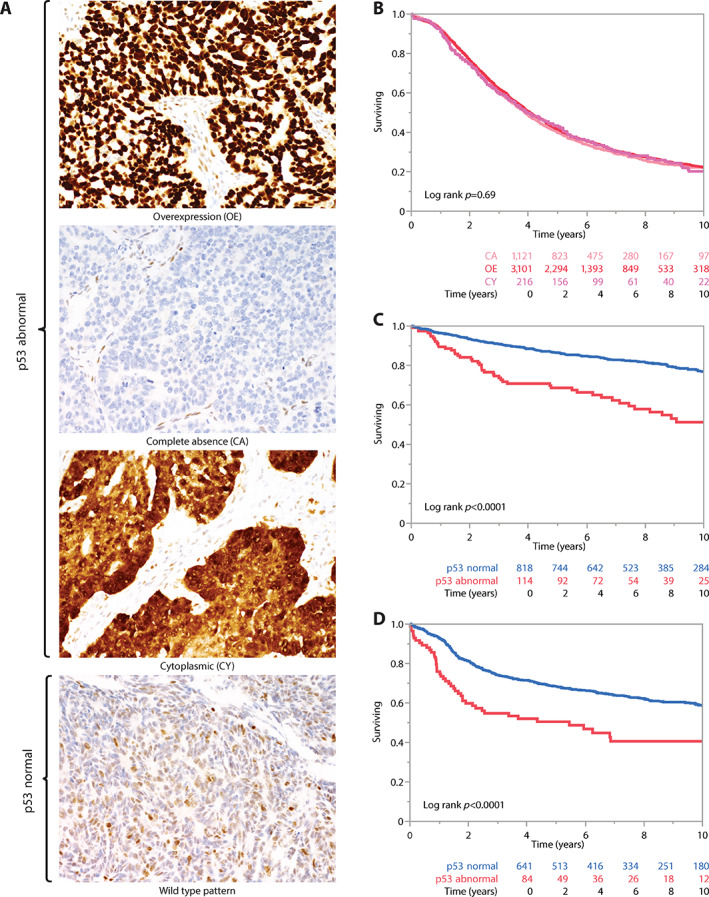
(A) p53 IHC patterns. (B) Kaplan‐Meier survival analyses of the three abnormal p53 IHC patterns in HGSC. (C) Kaplan‐Meier survival analyses of abnormal p53 versus normal p53 in EC. (D) Kaplan‐Meier survival analyses of abnormal p53 versus normal p53 in CCC.

HGSC showed the highest prevalence (4,630/4,957, 93.4%) of abnormal p53 staining compared to EC (116/973, 11.9%) and CCC (86/748, 11.5%). The distribution of different abnormal staining patterns was not significantly different across histotypes (Table 1). In HGSC, abnormal OE indicative of nonsynonymous/missense GOF mutations was the most common pattern (69.6%), followed by CA representing LOF mutations (25.4%) and the uncommon CY pattern reflecting mutations affecting the nuclear localisation of p53 (5.0%) (Table 1, Figure 1A).

### Abnormal p53 IHC patterns and univariate associations with overall survival, clinicopathological parameters, and selected biomarkers in HGSC

For HGSC, the frequency of normal p53 expression was higher than expected compared to previous smaller studies (current 6.6% versus expected 2–4%), suggesting possible misclassification of p53‐normal non‐HGSC as ‘HGSC’ in the current study [3, 26]. Therefore, analyses for HGSC were restricted to cases with abnormal p53 expression. Kaplan‐Meier survival analyses of 4,435 HGSC with follow‐up data showed overlapping survival curves for the three abnormal p53 patterns (log‐rank *p* = 0.69; Figure 1B). Univariate associations with clinical parameters such as age, stage, and residual disease did not show any significant differences across the abnormal p53 patterns (Table 2). No significant differences across selected biomarkers such as CD8+ TILs, CDKN2A, and germline *BRCA1/2* mutation status were observed.

**Table 2:** Clinicopathological characteristics and selected biomarkers across abnormal p53 expression patterns in HGSC

		Abnormal OE	%	Abnormal CA	%	Abnormal CY	%	Total	%	*P* value
HGSC	Total	3,221		1,176		233		4,630		
Age	Mean	60.5		60		61.2				0.121
	Range	26–93		28–91		35–87				
Stage	I/II	583	19.2	207	19.0	42	19.7	832	19.1	0.968
	III/IV	2,461	80.8	884	81.0	171	80.3	3,516	80.9	
	Unknown	177		85		20		282		
Residual disease	Absent	580	37.5	214	38.0	36	38.3	830	37.7	0.255
	Present	965	62.5	349	62.0	58	61.7	1,372	62.3	
	Unknown	1,676		613		139		2,428		
CD8+ TILs	Negative	386	15.7	148	16.5	26	14.3	560	15.8	0.818
	Low	385	15.7	154	17.2	28	15.4	567	16.0	
	Moderate	1,068	43.5	385	43.0	84	46.2	1,537	43.5	
	High	618	25.2	208	23.2	44	24.2	870	24.6	
CDKN2A	Normal	874	32.6	329	33.7	52	26.3	1,255	32.5	0.102
	Abnormal block	1,637	61.0	601	61.6	135	68.2	2,373	61.5	
	Abnormal absent	171	6.4	46	4.7	11	5.6	228	5.9	
*BRCA1/2*	Mutated	197	24.9	70	24.1	11	22.0	278	24.6	0.880
	Wild type	594	75.1	220	75.9	39	78.0	853	75.4	

*P* values calculated excluding cases with ‘unknown’ information.

### Binary p53 IHC status and uni‐ and multivariate associations with overall survival, clinicopathological parameters, and selected biomarkers in EC and CCC

Analyses were grouped for a binary comparison of cases with abnormal versus normal p53 IHC separately for EC and CCC [27]. Kaplan‐Meier survival analyses showed a significantly different overall survival between the groups for both EC and CCC (log‐rank *p* < 0.0001 for both; Figure 1C,D). The 5‐YSR for p53‐abnormal EC was 69% compared to 87% for p53‐normal EC, while the 5‐YSR for p53‐abnormal CCC was 50% compared to 68% for p53‐normal CCC.

**Figure 2:**
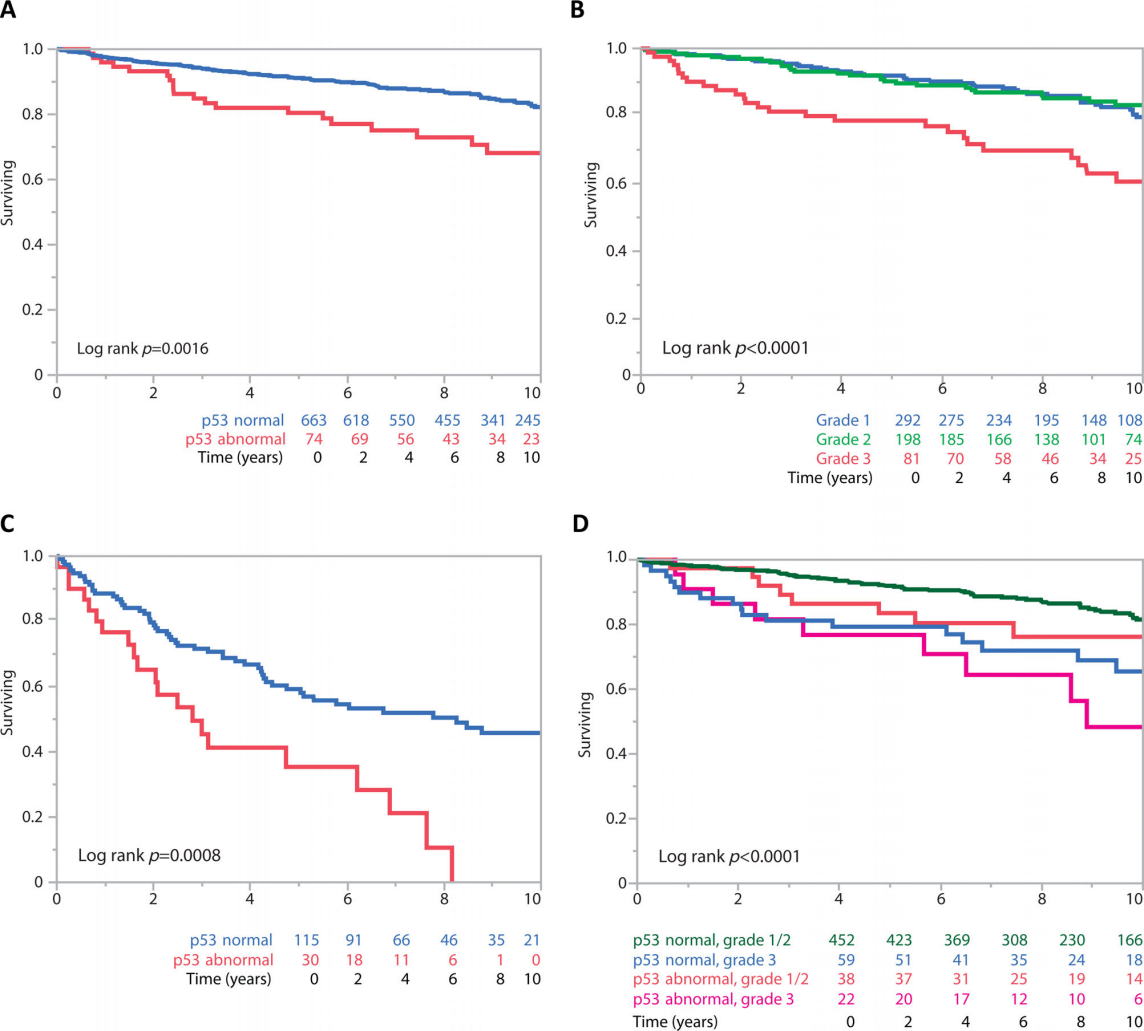
EC: stage‐stratified Kaplan‐Meier survival analyses. (A) By abnormal versus normal p53 at low FIGO stage (I/II) without residual disease. (B) By grade at low FIGO stage (I/II) without residual disease. (C) Abnormal versus normal p53 at high FIGO stage (III/IV). (D) By combined grade and abnormal versus normal p53 at low FIGO stage (I/II) without residual disease.

For EC, univariate survival analyses showed significant associations for stage, residual disease, binary p53 IHC status, grade, and age (supplementary material, Table S2). Except residual disease, these parameters remained significant in multivariate analysis (supplementary material, Table S2). Abnormal p53 IHC showed an HR of 2.18 (95% CI 1.36–3.47, *p* = 0.0011) after adjustment for age, stage, residual disease, grade, and stratified by OTTA study (supplementary material, Table S2). A Kaplan‐Meier survival analysis for stage I/II EC cases without residual disease showed p53‐abnormal cases having a 5‐YSR of 80% versus 91% for p53‐normal cases (log‐rank *p* = 0.0016; Figure 2A). In this low‐stage setting, the univariate HR for p53‐abnormal EC was 2.14 (95% CI 1.32–3.47, *p* = 0.0021, *n* = 737) compared to the reference group of p53‐normal EC, and the univariate HR was 2.59 for grade 3 (95% CI 1.65–4.07, *p* < 0.0001, *n* = 571) compared to the reference group grade 1 and 2 combined (Figure 2B). Stage III/IV cases with p53 abnormalities had a lower 5‐YSR (35%) than those who were p53 normal (58%, log‐rank *p* = 0.008, Figure 2C). With respect to clinical parameters, significantly more p53‐abnormal cases were diagnosed at a higher stage (27.6% stage III/IV for p53‐abnormal cases compared to 12.4% for p53‐normal cases, *p* < 0.001; Table 3). However, there were no associations with age or residual disease. With respect to biomarkers, p53‐normal EC cases demonstrated higher proportions of CD8+ TILs. Conversely, p53‐abnormal EC cases more commonly showed abnormal CDKN2A expression patterns and were commonly grade 3. However, slightly more than half of p53‐abnormal EC were diagnosed as low grade (grade 1 or 2). Approximately a third of grade 3 EC were p53 abnormal (32.5%, *n* = 41/126). Hence, there was roughly the same number of p53‐normal grade 3 EC cases (*n* = 85) compared to p53‐abnormal EC of all grades (*n* = 88). To assess the prognostic relation between p53 status and grade in EC, we created a combined variable for cases with available grade information (75.3%, 733/973): 76.4% (560/733) of cases were p53 normal and low grade (grade 1/2), 11.6% (85/733) were p53 normal and grade 3, 6.4% (47/733) were p53 abnormal and low grade, and 5.6% (41/733) were p53 abnormal and grade 3. Next, we performed a Kaplan‐Meier survival analysis for these four groups restricted to stage I/II EC cases without residual disease. Patients with p53‐normal, low‐grade EC had a 5‐YSR of 91.9%, compared to 83.5% for p53‐abnormal, low‐grade EC, 79.3% for p53‐normal, grade 3 EC, and 76.8% for p53‐abnormal, grade 3 EC (Figure 2D).

**Table 3:** Clinicopathological characteristics and selected biomarkers across binary p53 expression status in EC and CCC

		p53 abnormal	%	p53 normal	%	Total	%	*P* value
EC	Total	116		857		973		
Age	Mean	55		54.3		54.4		0.597
	Range	22–86		21–91		21–91		
FIGO Stage	I/II	76	72.4	695	87.6	771	85.9	<0.001
	III/IV	29	27.6	98	12.4	127	14.1	
Residual disease	Absent	51	44.0	371	43.3	422	43.4	0.697
	Unknown	57	49.1	442	51.6	499	51.3	
	Present	8	6.9	44	5.1	52	5.3	
CD8+ TILs	Negative	26	30.6	173	26.2	199	26.7	0.026
	Low	25	29.4	118	17.9	143	19.2	
	Moderate	24	28.2	259	39.2	283	38.0	
	High	10	11.8	110	16.7	120	16.1	
CDKN2A	Normal	40	41.2	619	82.1	659	77.4	<0.001
	Abnormal block	40	41.2	36	4.8	76	8.9	
	Abnormal absent	17	17.5	99	13.1	116	13.6	
Grade	I/II	47	53.4	560	86.8	607	82.8	<0.001
	III	41	46.6	85	13.2	126	17.2	
CCC	Total	86		662		748		
Age	Mean	56.1		55.2				0.452
	Range	28–83		27–84				
Stage	I/II	50	61.0	500	78.6	550	76.6	<0.001
	III/IV	32	39.0	136	21.4	168	23.4	
Residual disease	Absent	32	37.2	292	44.1	324	43.3	<0.001
	Unknown	33	38.4	305	46.1	338	45.2	
	Present	21	24.4	65	9.8	86	11.5	
CD8+ TILs	Negative	20	29.4	266	50.3	286	47.9	<0.001
	Low	12	17.6	122	23.1	134	22.4	
	Moderate	21	30.9	81	15.3	102	17.1	
	High	15	22.1	60	11.3	75	12.6	
CDKN2A	Normal	32	41.0	381	67.1	413	63.9	<0.001
	Abnormal block	34	43.6	70	12.3	104	16.1	
	Abnormal absent	12	15.4	117	20.6	129	20.0	

**Figure 3:**
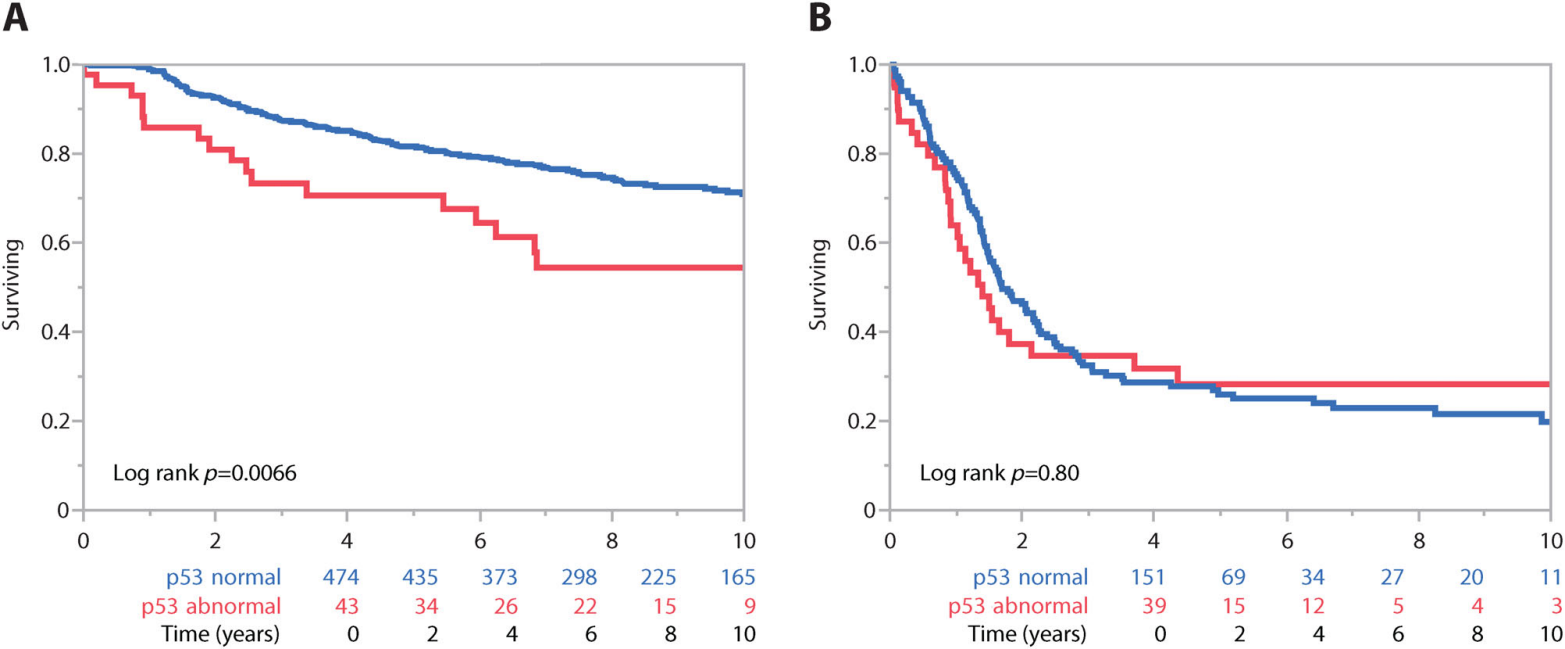
CCC: stage‐stratified Kaplan‐Meier survival analyses. (A) Abnormal p53 versus normal p53 at low FIGO stage (I/II) without residual disease. (B) Abnormal p53 versus normal p53 at high FIGO stage (III/IV).

For CCC, univariate analyses showed significant associations of overall survival with stage, age, residual disease, and binary p53 IHC status (supplementary material, Table S2). Stage, residual disease, and binary p53 IHC status remained significant in multivariate analysis (supplementary material, Table S2). Abnormal p53 IHC showed an HR of 1.57 (95% CI 1.11–2.22, *p* = 0.012) adjusted for age, stage, and residual disease and stratified by OTTA study (supplementary material, Table S2). A Kaplan‐Meier survival analysis for stage I/II CCC cases without residual disease revealed significant survival differences with p53‐abnormal cases having a 5‐YSR of 71% versus 82% for p53‐normal cases (log‐rank *p* = 0.0066; Figure 3A). No significant differences were observed for stage III/IV disease (Figure 3B). Univariate associations with clinical parameters showed that significantly more p53‐abnormal cases were diagnosed at a higher stage (39.0% stage III/IV for p53‐abnormal CCC compared to 21.4% for p53‐normal CCC, *p* < 0.001) and more likely to have residual disease after debulking surgery (Table 3). However, there were no associations with age. With respect to biomarkers, p53‐abnormal CCC cases showed higher proportions of CD8+ TILs and more commonly abnormal CDKN2A expression patterns.

### Correlation of p53 IHC patterns with *TP53* mRNA expression

**Figure 4:**
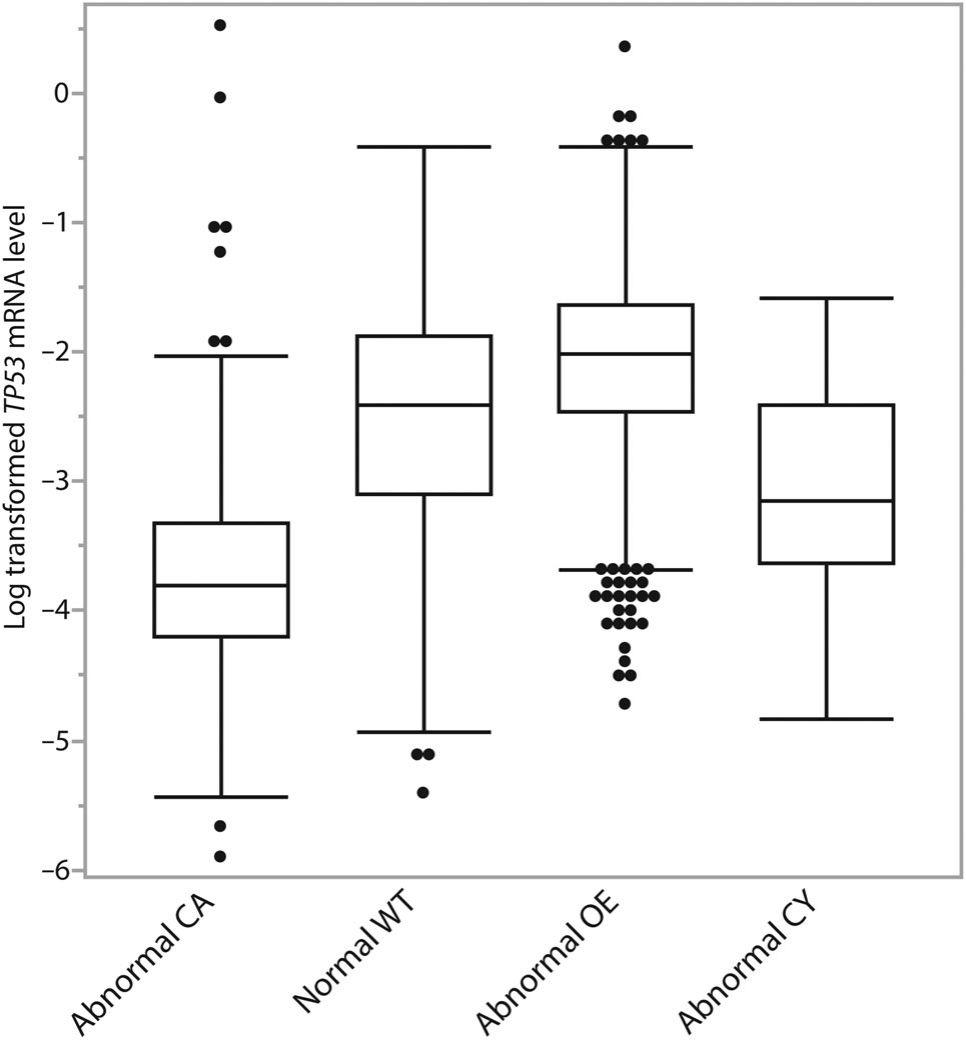
Association of p53 IHC patterns with *TP53* mRNA expression, including all histotypes.

Combining all three histotypes, there was a significant association of *TP53* mRNA expression with the four p53 IHC patterns for 2,111 analysed cases (*p* < 0.0001; Figure 4). The relative mean *TP53* mRNA expression normalised to housekeeping genes was (−2.54). Cases with abnormal p53 OE showed the highest mRNA expression (−2.06), followed by cases with normal p53 pattern (−2.56), while cases with abnormal CY (−3.09) and abnormal CA (−3.74) showed the lowest *TP53* mRNA expression.

## Discussion

Our study provides further evidence that the functional groups of *TP53* mutations assessed by abnormal surrogate p53 IHC patterns are not associated with survival in HGSC. For endometriosis‐associated ovarian carcinomas, we validate that abnormal p53 IHC is a strong independent prognostic marker for EC and demonstrate for the first time an independent prognostic association of abnormal p53 IHC with overall survival in CCC, especially for stage I/II CCC.

There is controversy on the prognostic role of the types or functional groups of *TP53* mutations in HGSC. Our data are in line with most of the studies that did not find differences between GOF and LOF *TP53* mutations in HGSC [6, 8, 9]. However, the results contradict our own earlier report that suggested an adverse prognostic association for the LOF surrogate pattern CA, although the association was only significant in one of three cohorts and the combined cohort [5]. The differences may be due to increased power in the current study (4,630 versus 502) and improved interpretation of p53 IHC with better delineation of abnormal patterns. The earlier study did not report the CY pattern and had a higher rate of the normal staining (12.0% versus 6.6% in the current study). The frequency of normal staining observed in the current study (6.6%) was still higher than expected: 2–4% of HGSC can show the normal/wild type p53 pattern by IHC due to late truncating *TP53* mutations that do not result in nonsense‐mediated mRNA decay of *TP53* [3, 26]. Poor antigenicity due to less standardised processing and longer ischemia times historically could have led to false negative interpretations of abnormal OE as a normal pattern in older specimens. However, since we could not exclude the possibility of some misclassified p53‐normal non‐HGSC, we restricted all HGSC analyses to abnormal patterns. In contrast to a previous study [28], we saw no difference across the abnormal p53 IHC patterns with respect to clinical parameters or selected biomarkers including *BRCA1/2* germline mutations. *TP53* mutations are a ubiquitous driver in HGSC and may be permissive for the development of a diverse range of mutational processes driving CIN in HGSC [29]. The prognosis is primarily determined by the extreme CIN with some prognostic effects from homologous recombination DNA repair deficiency, focal amplifications, or the tumour microenvironment [19, 30, 31, 32]. Given the very high prevalence of CIN in HGSC irrespective of type of *TP53* mutation, it is not surprising that p53 IHC patterns *per se* have no prognostic effects [29]. Since the development of p53 autoantibodies is associated with GOF mutations and decreased p53 protein degradation, we expected a higher CD8‐mediated immune response in HGSC with the abnormal OE pattern [33]. However, we did not observe an association between abnormal OE and CD8+ TILs. The expected association of p53 IHC patterns with *TP53* mRNA expression serves as cross‐validation of the assays, particularly, the low mRNA expression of the CA group with LOF mutation due to nonsense‐mediated decay of *TP53* mRNA. In a previous study, *TP53* mRNA expression was not associated with overall survival in HGSC (HR = 0.98, 95% CI 0.95–1.02, *p* = 0.57) [23].

For EC, our results are comparable to three earlier studies validating the unfavourable association of abnormal p53 IHC with overall survival [10, 11, 12]. Particularly clinically relevant is our finding in support of Krämer *et al* that abnormal p53 IHC can identify patients with low‐stage (I/II) disease at higher risk of death. These women require adjuvant therapy, likely in the form of platinum‐based chemotherapy, which is effective in women with p53‐abnormal endometrial ECs [17]. This would potentially render p53 not only a prognostic but also a predictive marker in EC. However, p53 IHC does not identify all women with low‐stage (I/II) disease at an increased risk of mortality. In contrast to the study by Krämer *et al*, we show that grade is also prognostic in the low‐stage setting. This difference between Krämer *et al* and our current study may be explained by interobserver variability in the assignment of grade [34] or by differences in the grading system used. Although we have no information on the specific grading system for individual cases, 95% of the EC cases in the current study were diagnosed between 1996 and 2011, during a time when the Silverberg grading system was the universal standard [35]. The Silverberg grading consists of a sum score of architectural complexity, degree of nuclear atypia, and mitotic count. In 2008, Malpica proposed a histotype‐specific grading system, and the FIGO grading system for endometrial ECs was adopted for use in ovarian ECs in the 2014 WHO classification [21, 36]. The FIGO/WHO grading ranks based on the percentage of solid architecture with the option of increase by one based on high nuclear atypia. Hence, relatively newer cases in the study by Krämer *et al* might have been graded by the WHO/FIGO system, which was not prognostic in the stage I/II setting. In line with this observation, Parra‐Herran *et al* recently showed a superior prognostic stratification by the Silverberg system, particularly for grade 3, compared to the WHO/FIGO system in EC [37]. Although p53‐abnormal EC is more likely grade 3, the majority of grade 3 EC are p53 normal, and both (p53‐abnormal, grade 3 EC and p53‐normal, grade 3 EC) have a similar but shorter survival compared to p53‐normal, low‐grade EC. The p53‐normal, grade 3 EC may include the recently described uncommon histotypes of dedifferentiated ovarian carcinomas or mesonephric‐like adenocarcinomas, which were historically diagnosed as EC and are associated with shorter survival [38, 39]. Excluding them would likely have resulted in even larger survival differences between p53‐abnormal and p53‐normal EC. It may be premature to dismiss grade, yet the limitations of interobserver reproducibility with grade are likely unresolvable. But future studies with large case numbers comparing Silverberg and FIGO/WHO grade could address this issue. Notably, in the setting of stage I/II without residual disease, the combination of p53 normal and low grade did not reach a 5‐YSR of 95% generally considered as threshold to withhold adjuvant chemotherapy [40]. In order to expand the spectrum of p53‐normal, low‐risk EC to substages beyond IA/IB, other biomarker combinations such as hormone receptor PR and CTNNB1 should be further validated [18, 20, 41, 42, 43, 44, 45].

Another interesting observation is the intermediate survival of p53‐abnormal, low‐grade EC. Because low‐grade tumours by Silverberg grading usually have low nuclear atypia, these tumours might not have developed CIN in the context of a *TP53* mutation, which was the rationale to use p53 as a surrogate for the copy number high genotype originally described by TCGA in endometrial carcinomas [46]. Future studies may refine the interplay between *TP53* mutations and copy number status in ovarian EC. A related question is then how to identify p53‐abnormal EC because the prevalence is relatively low and universal testing might not be justified. Perhaps, a similar approach of pathology‐driven selective testing based on nuclear features, which has shown high sensitivity in endometrial carcinoma, could be used [47].

For CCC, our study is the first to demonstrate that abnormal p53 status is an independent prognostic factor. Due to the relatively low prevalence of p53‐abnormal cases (12%) and the smaller effect compared to EC, large numbers were needed to show an independent prognostic association. Cunningham *et al* described two methylation clusters, whereby one was associated with a higher rate of *TP53* mutations and adverse clinical factors such as higher stage and residual disease, while the other cluster was characterised by *ARID1A/PIK3CA* co‐mutations, low‐stage disease, and aneuploidy [15]. Using targeted DNA and whole transcriptome RNA sequencing, Bolton *et al* also showed two distinct clusters with *TP53* and *ARID1A* as their respective lead alterations [16]. *ARID1A* mutations may appear relatively favourable, but this may be due to the inverse relationship with poor prognostic p53 because ARID1A by itself was not independently prognostic in a large series of CCC [48].

Both endometriosis‐associated ovarian carcinomas, EC and CCC, showed the same prevalence (12%) of abnormal p53 cases and similar prognostic associations. In both histotypes, abnormal p53 and abnormal CDKN2A often coexist. This finding should be taken into consideration when interpreting the association of block‐like p16 expression and shorter survival in EC and CCC [20]. However, differences between EC and CCC were also observed. In EC, the survival differences persisted in stage III/IV, while there were none in stage III/IV CCC, in line with the larger effect of p53 abnormal in EC compared to CCC in the multivariate model. Given the apparent efficacy of platinum‐based chemotherapy in p53‐abnormal endometrial ECs [17], abnormal p53 may also serve as a predictive marker for platinum‐based chemotherapy in ovarian EC while high‐stage p53‐normal EC may undergo alternative treatments (e.g. immune check point blockade for mismatch repair deficient [MMRd] cases). In contrast, p53 does not predict chemotherapy response in the generally chemoresistant CCC [49]. Another difference between EC and CCC is the change in directionality for the association of abnormal p53 status and CD8+ TILs. p53‐normal ECs have higher levels of CD8+ TIL infiltration. p53‐normal EC includes ultra‐(*POLE*) mutated [*POLE*mut] and hypermutated (MMRd) molecular subtypes, which due to their increased expression of neoantigens attract more CD8+ TILs [48]. CCC has generally lower levels of CD8+ TILs compared to EC [19], which is related to the absence of immunogenic *POLE*mut and MMRd molecular subtypes in CCC [19, 50]. *TP53* mutated CCC shows enriched expression of genes involved in immune‐related pathways [16].

Limitations of our study include incomplete data annotations for some covariates such as grade. For EC, the molecular subtype (i.e*. POLE*mut or MMRd) status was not available. However, the frequency of the prognostically favourable *POLE*mut is three times lower in ovarian EC (3.5%) compared to endometrial carcinomas (~10%) [11]. According to León‐Castillo *et al*, p53 abnormalities might be secondary to *POLE*mut; however, most are subclonal [51]. Truncal p53 abnormalities in the context of *POLE*mut were only rarely seen in 1 of 177 (0.6%) endometrial carcinomas [24]. Considering that *POLE*mut is three times less common in ovarian compared to endometrial EC, the possibility of co‐occurrence of truncal *TP53* and *POLE* mutations in ovarian EC is remote. Our data suggest that there is misclassification of p53‐normal non‐HGSC into the HGSC category; however, we restricted analyses to p53‐abnormal HGSC. On the other hand, we used WT1 alone or in combination with p53 to ensure that there was no misclassification of HGSC as ‘EC or CCC’. Additional overlay with *TP53* mutations would have strengthened the findings and potentially validated whether certain GOF mutations as identified by Tuna *et al* are associated with an unfavourable outcome in HGSC [9].

In conclusion, we provide strong evidence that abnormal p53 by IHC is an independent unfavourable prognostic marker in ovarian EC and CCC, which in conjunction with other biomarkers could inform clinical management in the low‐stage setting. Abnormal patterns of p53 IHC show no association with survival in HGSC. Hence, in HGSC, its role remains in the diagnostic and not the prognostic realm.

## Author contributions statement

MK and JDB conceived, designed, and supervisedthe study. PFR, EYK and MK collected immunohistochemical protein scores. MK and AW performed analyses. MK and EYK drafted the manuscript and JDB revised the manuscript. All authors contributed through collection, curation, and maintenance of respective consortia based, or local institution, collections of patient samples including recruitment and consenting of patients, clinical care, abstraction of clinical data, and updating of outcome and follow‐up data. All authors revised the manuscript and approved submission of the final version.

## Disclosures

JDB is a founder of Tailor Bio.

## Supporting information


**Table S1.** Study information
**Table S2.** Uni‐ and multivariate Cox regression analyses for EC and CCC

## Data Availability

Individual patient data and related tumor information underlying this article cannot be shared publicly due to data privacy protection laws. However, grouped data will be shared on reasonable request to the corresponding author.
